# A New Role for Plastid Thioredoxins in Seed Physiology in Relation to Hormone Regulation

**DOI:** 10.3390/ijms221910395

**Published:** 2021-09-27

**Authors:** Guillaume Née, Gilles Châtel-Innocenti, Patrice Meimoun, Juliette Leymarie, Françoise Montrichard, Pascale Satour, Christophe Bailly, Emmanuelle Issakidis-Bourguet

**Affiliations:** 1 CNRS, INRAE, Institute of Plant Sciences Paris-Saclay (IPS2), Université Evry, Université Paris-Saclay, F-91405 Orsay, France; neeg@uni-muenster.de (G.N.); gilles.chatel-innocenti@universite-paris-saclay.fr (G.C.-I.); 2 CNRS, Laboratoire de Biologie du Développement, Sorbonne Université, F-75005 Paris, France; patrice.meimoun@upmc.fr (P.M.); juliette.leymarie@u-pec.fr (J.L.); 3 IRHS-UMR1345, INRAE, Institut Agro, SFR 4207 QuaSaV, Université d’Angers, F-49071 Beaucouzé, France; francoise.montrichard@univ-angers.fr (F.M.); pascale.satour@univ-angers.fr (P.S.)

**Keywords:** redox, non-photosynthetic organ

## Abstract

In Arabidopsis seeds, ROS have been shown to be enabling actors of cellular signaling pathways promoting germination, but their accumulation under stress conditions or during aging leads to a decrease in the ability to germinate. Previous biochemical work revealed that a specific class of plastid thioredoxins (Trxs), the y-type Trxs, can fulfill antioxidant functions. Among the ten plastidial Trx isoforms identified in Arabidopsis, Trx y1 mRNA is the most abundant in dry seeds. We hypothesized that Trx y1 and Trx y2 would play an important role in seed physiology as antioxidants. Using reverse genetics, we found important changes in the corresponding Arabidopsis mutant seeds. They display remarkable traits such as increased longevity and higher and faster germination in conditions of reduced water availability or oxidative stress. These phenotypes suggest that Trxs y do not play an antioxidant role in seeds, as further evidenced by no changes in global ROS contents and protein redox status found in the corresponding mutant seeds. Instead, we provide evidence that marker genes of ABA and GAs pathways are perturbed in mutant seeds, together with their sensitivity to specific hormone inhibitors. Altogether, our results suggest that Trxs y function in Arabidopsis seeds is not linked to their previously identified antioxidant roles and reveal a new role for plastid Trxs linked to hormone regulation.

## 1. Introduction

Reactive oxygen species (ROS) are continuously produced in seeds, from embryogenesis to germination. They are massively produced during desiccation and imbibition, and to a lower extent, during dry seed storage [1,2,3]. Recent advances in seed physiology have led to consider ROS as key players in seed germination and dormancy. Indeed, studies on sunflower, pea and *Arabidopsis thaliana* (Arabidopsis) demonstrate that ROS are essential for dormancy alleviation and promotion of germination [4,5,6,7,8]. However, when the tight balance between ROS production and detoxification is impaired, ROS can act as toxic compounds, leading to oxidation of a wide range of biomolecules associated with a decrease in germinative quality [1]. Thus, critical lower and higher limits of ROS levels in seeds restrict the cellular events associated with the completion of germination, leading to the concept of an “oxidative window” for germination [1] that has been validated in the Col-0 ecotype of Arabidopsis [9].

ROS have been proposed to control germination in multiple ways. At the cellular level, it was demonstrated in vivo that localized production of hydroxyl radicals induces an oxidization of cell wall polysaccharides, contributing to the increase in embryonic cell elongation potential as well as the weakening of the micropylar endosperm to promote germination [7]. At the molecular level, cross-talks including feedback regulations between the cell redox status and phytohormones such as abscisic acid (ABA) or gibberellins (GAs) occur in seeds. On one hand, ABA inhibits ROS production in seeds [10,11,12] while GAs stimulate their accumulation [13,14]. On the other hand, ROS inhibit ABA accumulation and promote GAs synthesis [10,12]. Therefore, seed dormancy and germination are controlled by a complex network where ROS and the two antagonistic hormones are major players [5,9]. Furthermore, a signaling loop between plastid, mitochondrion, and the nucleus was recently proposed to control essential aspects of seed physiology including redox and hormonal regulations [15,16,17].

Thioredoxins (Trxs) constitute a family of small ubiquitous thiol oxidoreductases with a large number of important cellular functions [18,19]. In plants, they were initially discovered for their regulatory function in photosynthesis [20], then, later studies extended their functions to antioxidant systems [21,22,23]. Among the 20 canonical Trx isoforms found in Arabidopsis, 10 isoforms are found in plastids. Based on their sequence homologies, they are divided in five sub-types: the f, m, x, y, and z types.

Past studies have evidenced that, in leaves, Trxs play a crucial role in adapting photosynthesis to fluctuating light conditions, and that the different plastid Trx types can be functionally distinguished [24,25]. The regulation of carbon metabolism is attributed to the f and m-types, while the x and the y-type isoforms have antioxidant functions, being very efficient substrates for several thiol specific peroxidases [20]. Nonetheless, in planta studies reveal that plastid Trxs are actors in a complex redox regulatory system that is still not fully elucidated [25]. Moreover, some studies have depicted a localization of plastid Trxs in heterotrophic organs such as roots and flowers, or tissues such as vessels, opening the possibility of additional unknown roles, apart from their classical function in the regulation of photosynthesis-related enzymes [26,27].

In seeds, previous work has shown that cytosolic Trxs are necessary to promote protein reserves mobilization through the reduction of storage proteins in wheat, rice, barley and *Medicago* *truncatula* (Medicago) [28,29,30,31,32]. Moreover, in Arabidopsis, several cytosolic/mitochondrial Trx isoforms have also been implicated in seed physiology [33,34,35]. For instance, mutants for Trx o1, the main Trx in the mitochondrial matrix, exhibit lower seed germination rates and vigor, especially after deterioration or in stress conditions [34,36]. To date, no study has addressed the possible roles of plastid Trxs in seeds.

Analysis of transcript levels show that Trx genes are differentially expressed in Arabidopsis tissues [37]. Among messenger RNAs encoding plastid Trxs, Trx y1 transcripts are the most abundant in dry seeds where they are predominantly expressed in comparison to other organs of Arabidopsis plants [37]. In vitro biochemical studies showed that the y-type Trxs are preferential substrates for the reduction of plastidial targets such as peroxiredoxin (PRX) Q [23], glutathione peroxidase (GPX1) [38] and methionine sulfoxide reductase (MSR B2) [39]. We also recently found that the plastid isoform of monodehydroascorbate reductase (MDHAR6), a key enzyme of the glutathione-ascorbate pathway, is specifically activated by y-type Trx [40]. Moreover, in its oxidized state, Trx y can be an efficient activator of plastid glucose-6-phosphate dehydrogenase (G6PDH1), an NADPH-generating enzyme catalyzing the first step of the oxidative pentose phosphate pathway (OPPP) [41]. This pathway is a major source of reducing power for NADPH-dependent reactions occurring in non-photosynthetic conditions (dark) or in heterotrophic organs or tissues [42]. Thus, the y-type Trxs are believed to play an antioxidant role whatever their redox state [41]. The involvement of Trx y2 in the preservation of leaf MSR capacity (reduction of oxidized Met in proteins) was demonstrated in vivo and a positive correlation between the activity of the MSR and seed longevity has been established in Arabidopsis and Medicago [43,44].

Because of the importance of oxidative and redox processes in seed biology it was therefore of particular interest to study whether Trxs y could play a role in the regulation of seed germination. Here, by characterizing the seed physiology of Arabidopsis T-DNA mutants for this specific sub-class of Trxs, we unveil new functions for these redox proteins in seeds, in relation to phytohormone regulations.

## 2. Results

### 2.1. Characterization of the Germination Behavior of y-Type Trxs Mutant Seeds

Previously described single mutant lines for the two y-type Trx isoforms (Trx y1 and Trx y2) [43,44] were crossed to obtain a double mutant line deficient in both Trx y1 and Trx y2. Effect of gene mutations by T-DNA insertion was verified for both y-type Trxs in seeds at the mRNA level by RT-QPCR, and single, as well as double, mutants were considered as loss of function (Figure S1A,B).

Compensation for the absence of y-type Trx by other types of Trx was also investigated, and no significant change in the expression level of any of the other plastid Trx genes was observed in the seeds of the double mutant *trx y1y2*, in comparison with the wild-type (WT) (Figure S1C). These results indicated that these loss-of-function mutant lines are reliable tools to investigate a specific role played by Trxs y in Arabidopsis seed physiology. WT and Trx y mutant seed batches were obtained simultaneously and characterized for their germination behavior.

**Figure 1:**
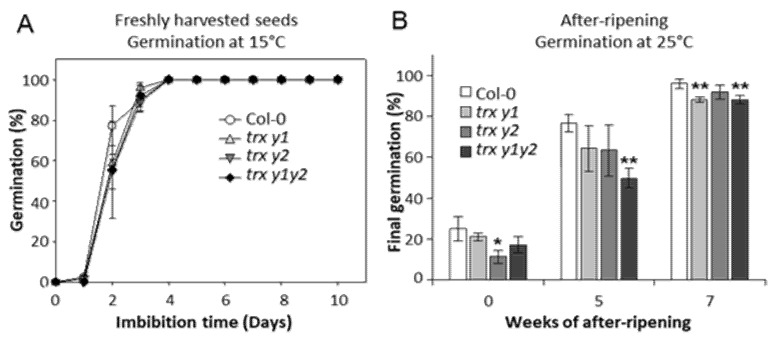
Germination of Trxs y mutant seeds at harvest and during after-ripening. Germination assays of Col-0 and Trxs y mutant seeds were performed in the dark with water-imbibed seeds. (**A**) Germination rates at 15 °C, directly after harvest. (**B**) Final germination percentage at 25 °C, after 10 days of imbibition of freshly harvested seeds, or after 5- and 7-weeks dry storage (after-ripening treatment). Means ± SD from triplicate experiments (3 independent seed batches) are shown. Significance levels: * *p* < 0.05, ** *p* < 0.01 compared to Col-0 by Student’s *t*-test.

We conducted germination assays and scored germination at regular intervals, using freshly harvested seeds, and 5- or 7-week after-ripened seeds (Figure 1 and Figure S2). Since light is a dormancy release factor [45], all germination assays were carried out in the dark to reveal dormancy [9,46]. All freshly harvested WT and mutant seeds (*trx y1* and *trx y2* single mutants and *trx y1y2* double mutant) fully germinated at 15 °C within 4 days with very similar rates (Figure 1A), indicating that their viability and their germinative quality were not affected by the mutation(s). In agreement with a previous report [9], at 25 °C, freshly harvested Arabidopsis seeds (Col-0 background) showed a very low germination percentage indicating that they were dormant. Only about 25% and less than 20% of the WT and mutant seeds germinated within 10 days, respectively (Figure 1B and Figure S2A).

Interestingly, after 5 weeks of after-ripening (dry storage), the WT seeds displayed 76.6 ± 4.1% final germination while it was significantly lower for the *trx y1y2* mutant, which germinated at only 49.8 ± 4.8%. Single mutants *trx y1* and *trx y2* displayed a less affected pattern than the double mutant line (Figure 1B and Figure S2B). After 7 weeks of dry storage, *trx y1* and *trx y1y2* mutants showed a significant slight reduction of their final germination after 10 days of imbibition at 25 °C (Figure 1B), and the initial germination speed (1–4 days imbibition) of all mutant seeds (single and double mutants) was clearly reduced compared to WT (Figure S2C).

**Figure 2:**
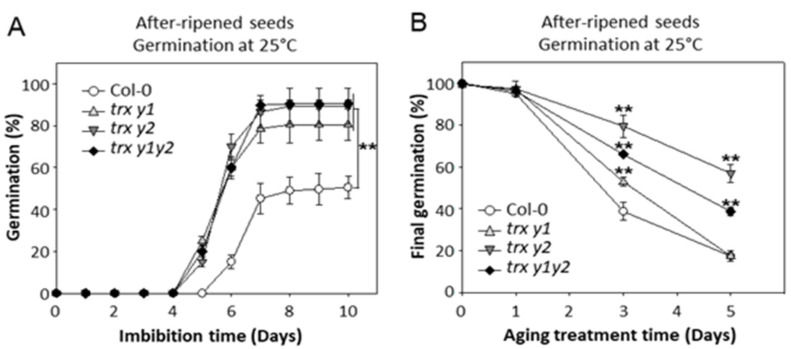
Germination of Trxs y mutant seeds under osmotic stress or after accelerated aging (AA). (**A**) After-ripened seeds were imbibed in the dark at 25 °C using a PEG solution (−0.6 MP) instead of water. (**B**) AA was performed by storing after-ripened seeds (after 12 weeks of dry storage) at 75% relative humidity for 1, 3, or 5 days at 40 °C. Then, seeds were dried back at 32% relative humidity and directly tested for germination capacity (same conditions as in Figure 1B). Means ± SD from triplicate experiments (3 independent seed batches). Significance levels: ** *p* < 0.01 compared to Col-0 by Student’s *t*-test.

We then investigated the germination capacity of fully after-ripened WT and y-type Trx mutant seeds at low water potential, by using a PEG solution (−0.6 MPa at 25 °C) instead of water for seed imbibition. In these conditions, germination of WT seeds was drastically affected, germinating to only *ca.* 50% after 10 days of imbibition (Figure 2A). In the same conditions, mutant seeds kept a remarkably higher germination rate of 80–90% (80.5 ± 7.5%, 89.2 ± 8.5% and 90.4 ± 1.6% for *trx y1*, *trx y2* and *trx y1y2* mutants, respectively).

We also compared the sensitivity of WT and mutant seeds to accelerated aging (AA), a method inducing massive oxidative damages [47]. AA was performed using fully after-ripened seeds for 1, 3, or 5 days. Seeds were dried, and their germination was scored at 25 °C (Figure 2). The treatment strongly decreased the germination percentage of Col-0 seeds from 100% to ca. 39% and 17% after 3 and 5 days of treatment, respectively (Figure 2B). In the same conditions, single and double Trx mutant seeds clearly showed a better tolerance to AA. After 3 days, the mutants had a final germination of ca. 79%, 66%, and 53% for the *trx y2*, *trx y1y2* and *trx y1* mutants, respectively. After 5 days of AA, the mutant seeds were still able to germinate up to *ca.* 57% for the *trx y2* and up to *ca.* 39% for the *trx y1y2* mutant while less than 20% of WT seeds germinated (Figure 2B).

Altogether, these data showed that in Arabidopsis seeds y-type Trxs are required (i) to fully integrate the effect of after-ripening, and (ii) to arrest germination, under water limitation at imbibition, and (iii) to tolerate aging.

### 2.2. Seed Redox Status Is Not Affected by y-Type Trx Mutations during Imbibition

To investigate if the above-described mutant seed phenotypes were linked to perturbation of the cellular redox balance, the global redox status of Trxs y mutant seeds was evaluated at the level of their ROS contents and their protein redox state. H_2_O_2_ levels and O_2_^−^ accumulation patterns were examined on freshly harvested seeds after 3 and 24 h imbibition at 25 °C in the dark. No significant changes in the H_2_O_2_ level (scopoletin oxidation assay) were found in mutant seeds compared to WT seeds at the two tested imbibition time points (Figure S3A). Similarly, the pattern of O_2_^−^ accumulation (nitroblue tetrazolium (NBT) staining) in seeds was not obviously perturbed in the absence of y-type Trxs, accumulation of NBT formazan being equivalently found at the radicle tip of WT and double mutant genotypes (Figure S3B).

**Figure 3:**
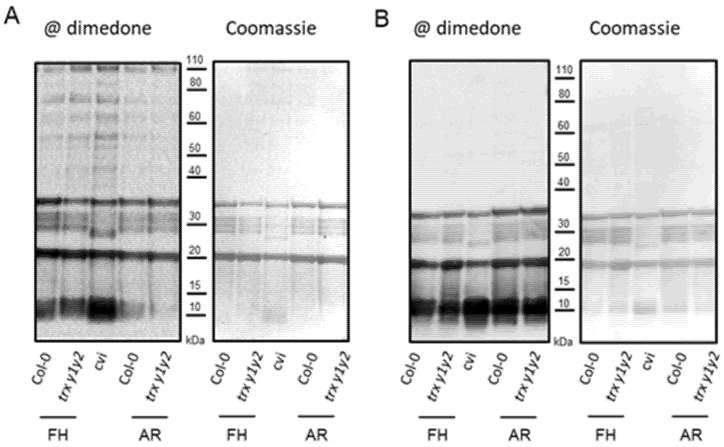
Protein cysteine oxidation in WT and *trx y1y2* mutant seeds. Seeds were imbibed either (**A**) with water, or (**B**) in oxidative stress-triggering conditions (in presence of ascorbate 10 mM + CuSO_4_ 10 µM) in the dark for 24 h. Oxidized protein Cys residues (sulfenic acid form) were specifically immuno-detected after a labeling treatment using dimedone, SDS-PAGE, and Western blot chemiluminescence technology (left panel). For comparison between samples, Coomassie blue staining of the corresponding membrane is shown (right panel). Freshly harvested (dormant) (FH) seeds or 8-week after-ripened seeds (non-dormant) (AR), as well as Cape Verde Island (cvi) seeds were analyzed.

In the leaves of the same *trx y* mutant lines used in the present study, we previously found that proteins were more oxidized compared to WT [40]. In addition, studies in sunflower and with Arabidopsis mutants have respectively shown that seed dormancy alleviation and perturbations of seed ROS homeostasis correlate with variations in protein carbonylation, a hallmark of protein oxidation [4,48,49]. Thus, we expected proteins to be more oxidized in the *trx y1y2* mutant seeds compared to WT seeds. By examining protein cysteine thiols (mBBr labeling) and protein carbonylation (oxyblot) levels, we were not able to evidence any clear difference between dormant and non-dormant seeds of WT and mutant seeds (Figure S4). In addition, we examined global protein cysteine oxidation using dimedone as a probe since it reacts with oxidized cysteines (the sulfenic acid, over further oxidation forms) in model proteins and cells. Western blot analysis using anti-dimedone antibodies revealed a strong protein Cys oxidation level in WT and *trxy1y2* mutant dormant seeds, as well as in seeds of the hyper-dormant ecotype Cape Verde Island (cvi) (Figure 3A).

The cysteine sulfenic acid signal was much lower in imbibed non-dormant seeds compared to dormant seeds, for both WT and mutant. We also imbibed seeds in the presence of ascorbate and copper to trigger free radical pro-oxidants (Figure 3B). As expected, with this treatment, protein Cys oxidation was strong in all seed samples analyzed, especially in after-ripened seeds. Again, no difference could be evidenced between Col-0 and *trxy1y2* seeds.

Previous studies have also shown that germination of mutant seeds with defects in antioxidant systems is impaired in presence of methylviologen (MV) [48,50]. Therefore, seed germination was tested in the presence of 10 µM MV. We observed that the germinative capacity of WT and mutant seeds was almost unaffected by this treatment, all genotypes showing a final germination percentage close to 100% after 9 days of imbibition (Figure S5). However, while germination of WT seeds was severely delayed, germination kinetics of mutant seeds were not affected (compare data from Figure S5 with germination in control conditions shown in Figure S2C).

Again, no evidence could be obtained about y-type Trxs playing a major role in the antioxidant capacity of seeds at germination. On the contrary, mutation(s) seemed to have a positive effect on the seed capacity to germinate under oxidative stress conditions.

We noticed that Trxs y mutant seeds manifested a delay in after-ripening compared to WT seeds (Figure 1B and Figure S2) similar, although less marked, to the reported phenotype for *rbohd* mutant seeds [9]. The *RBOH* genes encode NADPH oxidases catalyzing the production of apoplastic superoxide from oxygen and NADPH, probably responsible for the ROS burst promoting germination. Notably, we found that, compared to Col-0, RBOHD transcripts were depleted in the *trx y1y2* mutant seeds (Figure S6), in agreement with the shared phenotype with the corresponding mutant.

Overall, our data validated a role played by y-type Trxs in seed physiology but did not support their antioxidant function initially hypothesized in Arabidopsis seeds.

### 2.3. ABA Metabolism Is Impaired in y-Type Trx Mutant Seeds

ROS production acts in concert with hormone signaling to regulate seed germination, dormancy, and longevity [51,52,53,54,55,56]. Here, we found that mutants depleted in y-type Trxs are affected in these seed traits. Therefore, perturbations in hormone metabolism and signaling were investigated in the *trx y1y2* mutant seeds by analyzing abscisic acid (ABA) and gibberellin (GA) marker gene expression by RT-QPCR.

**Figure 4:**
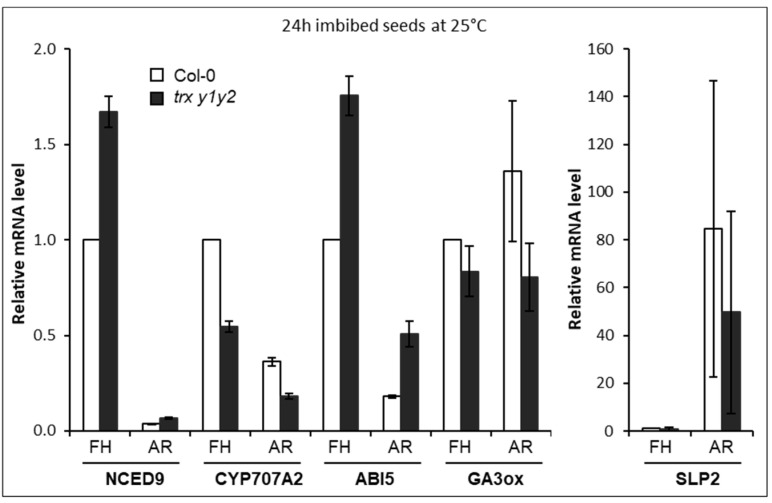
Expression of ABA and GA metabolism-related genes in *trx y1y2* mutant seeds. Transcript abundance of AtNCED9 (encoding a 9-*cis*-epoxycarotenoid dioxygenase involved in ABA synthesis [57]), AtCYP707A2 (encoding an 8′hydroxylase involved in ABA catabolism [58]), AtABI5 (encoding a transcription factor involved in ABA signaling [59]), AtGA3ox (encoding a gibberellin 3-beta-hydroxylase catalyzing the final step of the biosynthetic pathway of GAs [60]), and AtSLP2 (encoding a shewanella-like protein phosphatase, implicated in GA signaling [9]) was examined using RT-QPCR in freshly harvested (FH), or 7-week after-ripened (AR) seeds imbibed for 24 h in the dark at 25 °C. Transcript levels were normalized to the expression of At4g12590 seed constitutive gene and an arbitrary unit was assigned to the expression level in the Col-0 (FH) sample for each gene. Means ± SD from duplicate experiments.

In both WT and mutant seeds, after 24 h of imbibition at 25 °C in darkness, ABA marker genes were downregulated in after-ripened seeds compared to freshly harvested seeds (Figure 4). Compared to Col-0, *trx y1y2* mutant seeds displayed higher mRNA levels of AtNCED9 (ABA synthesis) and AtABI5 (ABA signaling). Conversely, AtCYP707A2 gene transcripts (ABA catabolism) were decreased twofold in freshly harvested mutant seeds compared to WT (Figure 4). As previously reported in Col-0 [9], we found that AtSLP2 transcripts (GA signaling) were strongly induced in both WT and mutant seeds when comparing freshly harvested to after-ripened seeds, while AtGA3ox1 gene (GA synthesis) expression level stayed globally constant in both genotypes (Figure 4).

Overall, ABA and GAs transcript marker perturbations were consistent with mutant germination phenotypes, suggesting that Trxs y function(s) in seeds may be related to the hormonal control of germination.

**Figure 5:**
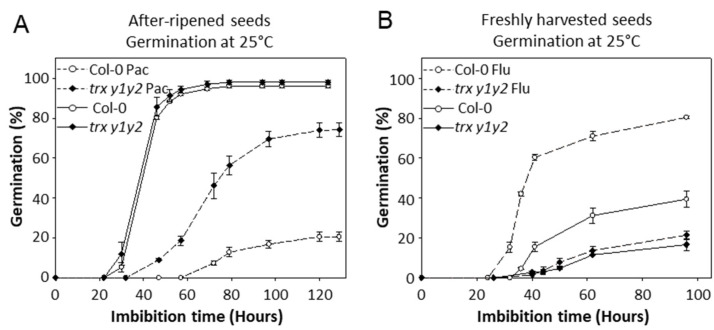
Germination of WT and *trx y1y2* mutant seeds in the presence of hormone synthesis inhibitors. (**A**) After-ripened seeds were germinated in the light with addition of paclobutrazol (biosynthesis inhibitor of GAs) (Pac, 5 µM). (**B**) Freshly harvested seeds were germinated in the dark with addition of fluridone (biosynthesis inhibitor of ABA) (Flu, 25 µM). Triplicates were averaged. Bars indicate SD of the mean.

To confirm this possibility, germination of Col-0 and *trx y1y2* mutant seeds was monitored in the presence of biosynthesis inhibitors of ABA (fluridone (Flu) [61,62]) or GAs (paclobutrazol (Pac) [63]). As expected, Pac inhibited the germination of after-ripened seeds of both genotypes, but *trx y1y2* mutant seeds were clearly less affected (25% inhibition) compared to Col-0 seeds (80% inhibition) (Figure 5A).

We found that Flu, at a concentration of 25 µM, allowed Col-0 seeds to germinate faster with a doubled final germination percentage while it failed to promote *trx y1y2* mutant seed germination (Figure 5B).

Clearly, depletion of Trxs y modified seed sensitivity to treatments directly affecting ABA and GAs metabolisms.

## 3. Discussion

Among the 10 Arabidopsis plastid Trxs, the transcripts encoding the Trx y1 isoform were found to be the most abundant in dry seeds [37] and those of the Trx y2 gene increased rapidly upon seed imbibition [23], suggesting a specific role for y-type Trxs in seed physiology. Here, we used y-type Trx single and double T-DNA mutant lines to investigate the presumed importance of this sub-class of Trxs in seed physiology.

While most past functional studies on chloroplastic Trxs were dedicated to their role in modulating photosynthesis [25,64], our study addressed their potential role in a non-photosynthetic organ (the seed) and in the absence of light, contrary to the vast majority of previous studies conducted in the leaf and under the light.

### 3.1. Plastid Trx-PRX Redox Couples Are Present in Arabidopsis Seeds

Biochemical studies have collectively attributed an antioxidant role to y-type Trxs [23,38,39,41]. Trxs cannot directly scavenge ROS. They play their antioxidant role through their function as reducing power transmitters to antioxidant enzymes such as peroxidases. Some of them, known as peroxiredoxins, mostly use Trxs as proton donors (PRXs and GPXs) for H_2_O_2_ and alkyl hydroperoxides reduction [65]. Notably, microarray data suggested that expression of some plastid PRX and GPX genes is high in developing embryos (GPXI and PRXIIE) as well as in dry and imbibed seeds (GPXI, PRXQ, PRXIIE, and 2-CYS PRXA) (Figure S7). At the protein level, known Trx y antioxidant targets such as PRX Q and GPX1 [23] were identified in Arabidopsis dry and 6 h imbibed seeds by proteomics, together with Trx y1 [66]. Thus, expression patterns indicate that plastid Trx-PRX redox couples are present in Arabidopsis seeds.

### 3.2. Trxs y Do Not Play a Major Role in Seed ROS Homeostasis

Past studies having shown that ROS promote the germination of dormant seeds [1,5,9,12], Trxs y mutant seeds were expected to display higher germination rates than the WT at harvest. High levels of ROS being also associated with seed aging [47,52,56], Trx mutant seeds were also expected to be prone to aging and sensitive to harsh conditions during germination. Instead, our results revealed phenotypes for Trxs y mutants opposite to expectations based on the antioxidant functions previously proposed for this Trx sub-class [23,38,39]. Compared to WT, mutant seeds required a longer period of dry storage to reach maximum germination ability (Figure 1 and Figure S2) and after-ripened seeds germinated better in adverse conditions (water limitation: Figure 2A; oxidative stress: Figure S5) and were more tolerant to accelerated aging (Figure 2B). The ability of y-type Trxs to mediate dithiol-disulfide exchanges with plastid PRXs, including PRX Q, was demonstrated by biochemical studies [23]. Of note, seeds of the PRX Q knock-out mutant were found to be less dormant [67] in accordance with the ROS scavenging role of the peroxiredoxin [23]. Moreover, in mutant seeds we did not find obvious changes in ROS contents (Figure S3), as well as in global protein redox status (Figure 3 and Figure S4), in comparison to WT seeds. A direct assessment of plastid PRX activities and a deeper investigation of the cellular redox status (i.e., pool size and reduction state of antioxidant metabolites such as glutathione, ascorbate, or NAD(P)H) in mutant seeds is still required to fully rule out a genuine role played by y-type Trxs in seed redox homeostasis. Nevertheless, our data collectively suggest that in seeds y-type Trxs may not be limiting for PRX regeneration or global antioxidant capacity since *prx q*, as well as other mutants affected in antioxidant systems, show opposite germination phenotypes in comparison with Trxs y mutants [9,48,67]. In Arabidopsis seeds, like previously proposed in leaves, plastid PRXs might be alternatively reduced by other Trx isoforms or by NTRC [68].

### 3.3. Trxs y May Be Involved in Redox Regulation of Metabolism at Seed Germination

Recent reports have refined our understanding of Trxs functions for chloroplast metabolism adjustments. Trxs act as a link between peroxidases that directly sense H_2_O_2_ and the activity of redox-regulated metabolic enzymes [69]. In leaves, it was recently shown that Trx y2 interacts with 2-Cys PRX for sensing ROS levels in the chloroplast while poorly contributing to H_2_O_2_ reduction, compared to other Trx isoforms such as Trx x or NTRC [70]. Since low or high levels of ROS promote or restrain germination, respectively, based on our data, it is tempting to propose that rather than participating in ROS detoxification, y-type Trxs may influence germination by integrating ROS levels and modulating metabolic enzymes in seed (pro)-plastids. By testing germination in the dark, we evidenced that Trxs y deficiency caused perturbation of seed dormancy. Interestingly, past studies supported the idea that activation of the OPPP is an early event in dormancy breakage [71,72]. In particular, G6PDH, a Trx-regulated enzyme in plastids, was proposed to be important for seed release from dormancy, and we previously showed that Trx y1 is an efficient G6PDH activator in vitro [41]. Thus, for future investigations, it will be of interest to explore whether Trxs redox regulation is important for enzyme activities linked to carbohydrate mobilization during Arabidopsis seed germination.

### 3.4. Trxs y Function in Seeds Is Linked with Hormones

Abscisic acid and gibberellins are considered as major contributing phytohormones for seed physiology. ABA is strictly required to induce and maintain dormancy during seed maturation and imbibition, respectively [53]. Our expression analysis of hormone marker genes (Figure 4) revealed ABA perturbations in seeds in the absence of Trxs y. ABA is also known as a key hormone controlling seed desiccation tolerance [54] and germination arrest in the context of unfavorable environmental conditions such as water deficit [55,73,74,75]. ABA is also considered as a major component for the acquisition of seed longevity during maturation [56]. In the present study, y-type Trx mutant seeds were found to be affected in all aforementioned seed traits and recalcitrant to germination promotion by Flu (Figure 5B). Furthermore, microarray data suggested a link between GAs synthesis and Trx y2 gene expression, since its strong induction at seed imbibition ([23], seed eFP browser) was compromised in WT seeds imbibed in the presence of Pac ([76], seed eFP browser), as well as in the *ga1-3* mutant where it could be restored by exogenous application of GA_4_ ([77], seed eFP browser). These GA-dependent effects at seed imbibition on Trx transcript levels seem to be specific to Trx y2, since Trx y1 gene expression was not affected in the *ga1-3* mutant and by GA-related pharmacology. Here, we found that after-ripened seeds of the *trx y1y2* mutant were less sensitive to Pac compared to Col-0 seeds (Figure 5A). This result is also suggesting a functional link between Trxs y and GA biosynthesis.

### 3.5. Trxs y May Regulate Hormone Synthetic Pathway

GAs, like ABA, are synthesized in plastids from a common precursor 1-deoxy-D-xylulose-5-phosphate (DXP). Enzymatic steps involved in isoprenoid precursor biosynthesis in plants are multi-level regulated, and redox dependency as well as regulation were proposed for some of them [78,79]. Interestingly, DXP reductoisomerase (DXR), catalyzing the conversion step of DXP to isopentenyl diphosphate (IPP), was trapped on a Trx affinity column and thus proposed as a new Trx target in plastids [80], which needs to be experimentally validated. Our results further suggest that Trxs of the y sub-class could be good candidates for a Trx-dependent DXR regulation in seeds.

In conclusion, in the present study, using a reverse genetics approach, we provided evidence that plastid Trxs of the y-type are important for seed physiology in multiple aspects. Trx y mutant seeds display remarkable traits, such as increased longevity and germination in conditions of reduced water availability, which are pivotal for agriculture. Thus, our work paves the way for future investigation of the specific roles of Trxs y for the potential improvement of genetic resources conservation and plant stress tolerance. At the molecular level, we could not correlate seed mutant phenotypes with initially suspected effects attributable to the known antioxidant functions of Trxs y. We rather found that Trxs y participate in signaling functions related to hormonal regulation in seeds. Based on the present knowledge, it is difficult to position the role(s) played by Trxs y in hormonal control set by redox regulations in direct relation to cell ROS homeostasis during seed germination. Future work will be dedicated to elucidating the molecular mechanism(s) underlying the connection between Trxs y and hormone control. Our present study indicates that involvement of y-type Trxs in seed physiology probably occurs through changes in ABA/GA-related functions. This is a new hypothesis for plastid localized Trxs, enlarging the spectrum of their functions beside their implication in the regulation of carbon metabolism and ROS detoxification.

## 4. Methods

### 4.1. Plant Materials and Seed Batches Production

Arabidopsis (*Arabidopsis thaliana*, ecotype Col-0) and the 2 Trx y deficient mutants (in Col-0 background) used in this study were previously isolated and described [40,43]. The double mutant *trx y1y2* was obtained by crossing the two homozygous mutants *trx y1* (At1g76760 gene, SALK_103154) and *trx y2* (At1g43560 gene, SALK_028065).

Plants were grown in a growth chamber with a 16 h photoperiod at a photon flux density of 200 µE m^−2^ s^−1^ at 22 °C 60% HR during daytime and 20 °C 55% HR during nighttime. Siliques were harvested at maturity 8 weeks after sowing and dried in the dark at 18 °C 55% HR for 10 days. For this study, all seed batches were obtained by cropping the plants concomitantly in the same controlled conditions. Freshly harvested seeds were either stored at −20 °C to preserve their dormancy [9,58] or after-ripened at 25 °C 30% HR in the dark.

### 4.2. Germination Assays

All germination assays were performed by placing seeds in Petri dishes (100 seeds per dish, three replicates), on a filter paper on top of a layer of cotton wool moistened with de-ionized water, except for tests in the presence of hormone inhibitors where seeds were germinated on ½ MS agar medium (fluridone or paclobutrazol, at 25 and 5 µM final concentration, respectively). In some experiments, water was replaced by PEG solution (−0.6 MP) or methylviologen (10 µM). Assays were performed in darkness (except germination in the presence of Pac) at a controlled temperature of 15 or 25 °C (as specified). The percentage of germinated seeds was scored under a lens daily, up to 5–10 days. Radicle protrusion through the envelopes was taken as the criterion for germination.

### 4.3. Seed Accelerated Aging Treatment (AA)

AA was performed according to Hay et al. [81]. Seeds were placed in open tubes at 75% relative humidity (HR) at 40 °C for 1, 3, or 5 days. Then, seeds were dried back to their initial moisture content (*ca.* 5%) at 20 °C and directly used for germination assays.

### 4.4. Determination of Hydrogen Peroxide Production

H_2_O_2_ production–diffusion was monitored by measuring the decrease in scopoletin fluorescence in the incubation medium for 1 h, using 24 h imbibed seeds at 25 °C. A 30 mg aliquot of seeds was incubated in 250 µL of potassium phosphate buffer (20 mM, pH 6.0) containing 5 mM scopoletin (Sigma-Aldrich, Saint Quentin-Fallavier, France) and 1 U mL^−1^ (final concentration) horseradish peroxidase (Roche Diagnostics, Meylan, France) in darkness at 25 °C on a shaker, as described by Schöpfer et al. [13]. H_2_O_2_ production was evaluated by the decrease in fluorescence (excitation, 346 nm; emission, 455 nm) of the incubation medium and was transformed into molar H_2_O_2_ concentration using a linear calibration curve. Results were expressed per mg of the initial fresh weight of dry seeds.

### 4.5. Total RNA Extraction and Transcript Level Analysis

A 30 mg (dry weight) aliquot of seeds was ground in liquid nitrogen with 5% (w/v) polyvinylpyrrolidone and total RNA extraction was performed using the SV Total RNA Isolation System #Z3100 (Promega, Charbonnières, France), following the manufacturer instruction except that the centrifugation to separate lysate from soluble fraction was replaced by an ultracentrifugation of 15 min at 60,000 rpm (Rotor Beckman TLA 122) to separate lipids. The reverse transcription step was performed with up to 480 ng total RNA, using the ImProm-II™ Reverse Transcription System #A3802 (Promega, Charbonnières, France) with oligo(dT) primer. Gene expression profiling was conducted by real-time quantitative RT-PCR using triplicate reactions for each sample and a gene-specific primer pair previously specified [9,37,82]. Reactions were assembled with 5 ng cDNA template, 250 nM of gene-specific forward and reverse primers, and 7.5 μL SYBR Green reagent (Roche Diagnostics, Meylan, France) in a total volume of 50 µL in 96-well plates sealed with optical film. Reactions were conducted using a LightCycler^®^ 480 Real-Time PCR System (Thermo Fisher Scientific, Les Ulis, France). The amplification protocol was as follows: activation at 95 °C for 10 min, 45 or 55 cycles of amplification at 95 °C for 15 s, 60 °C for 20 s, 72 °C for 15 s, and then 95 °C for 30 s. Melting curves were obtained after each run starting from 65 to 95 °C to confirm that single, specific products were produced. The results were standardized by comparing the data to reference gene At4g12590, a constitutively expressed gene in seed [82], except for Figures S1 and S2 where standardization was made using the PP2A gene (At1g13320, a constitutively expressed protein phosphatase 2A [83]). The quantification of gene expression was performed using the comparative CT method [84]. An arbitrary value of 1 was assigned to the 25 °C and 24 h imbibed Col freshly harvested seed samples, which were used as control samples for normalization [85], except in Figures S1 and S2 where normalization was done on Col dry freshly harvested seed.

### 4.6. Global Protein Redox Status

Freshly harvested or after-ripened seeds were imbibed for 24 h at 25 °C, either with water or with ascorbate and copper (ascorbate 10 mM plus CuSO_4_ 10 µM) to trigger cysteine oxidation to sulfenic acid (RSOH) by ROS [86]. RSOH in seed proteins was detected after chemical derivatization with 5,5-dimethyl-1,3-cyclohexanedione (dimedone) forming a stable thioether adduct that could be immunodetected. Protein samples (50 µg) were separated by 4–20% SDS-PAGE. Following electrophoresis, proteins were transferred onto a PVDF membrane and detected using 1:10,000 dilution of a rabbit anti-cysteine sulfenic acid (Merck Millipore, Guyancourt, France) antibody and 1:20,000 diluted anti-rabbit horseradish peroxidase (HRP)-conjugated secondary antibody (GE Healthcare, Tremblay-en-France, France), and finally, oxidized proteins were visualized by chemiluminescence (GE Healthcare, Tremblay-en-France, France). Seed protein thiols and carbonyls were detected as previously described [31,87].

### 4.7. Statistics and Software

Statistical analyses were performed using the Student’s *t*-test function in SigmaPlot 12.0.

## Data Availability

The data presented in this study are available on request from the corresponding authors.

## Supplementary Materials

The following are available online at https://www.mdpi.com/article/10.3390/ijms221910395/s1, Figure S1. Transcript levels of Trxs genes in *trx y* mutant seeds, Figure S2. Germination of *trx y* mutant seeds along after-ripening, Figure S3. Hydrogen peroxide levels and superoxide anion accumulation patterns in *trx y* mutant seeds, Figure S4. Redox status of seed proteins in Col-0 and the *trx y1y2* mutant, Figure S5. Germination of *trx y* mutant seeds under oxidative stress, Figure S6. Expression level of *RBOHD* gene in the *trx y1y2* mutant seeds, Figure S7. mRNA levels of peroxiredoxins (plastid isoforms) in Arabidopsis.
