# Supplementary material for: A New Role for Plastid Thioredoxins in Seed Physiology in Relation to Hormone Regulation
Source: Int J Mol Sci. 2021 Sep 27;22(19):10395. doi: 10.3390/ijms221910395 (PMC8508614; doi:10.3390/ijms221910395)
Supplement: Supplementary file 1 [file ijms-22-10395-s001.zip › ijms-1376939-supplementary.pdf]

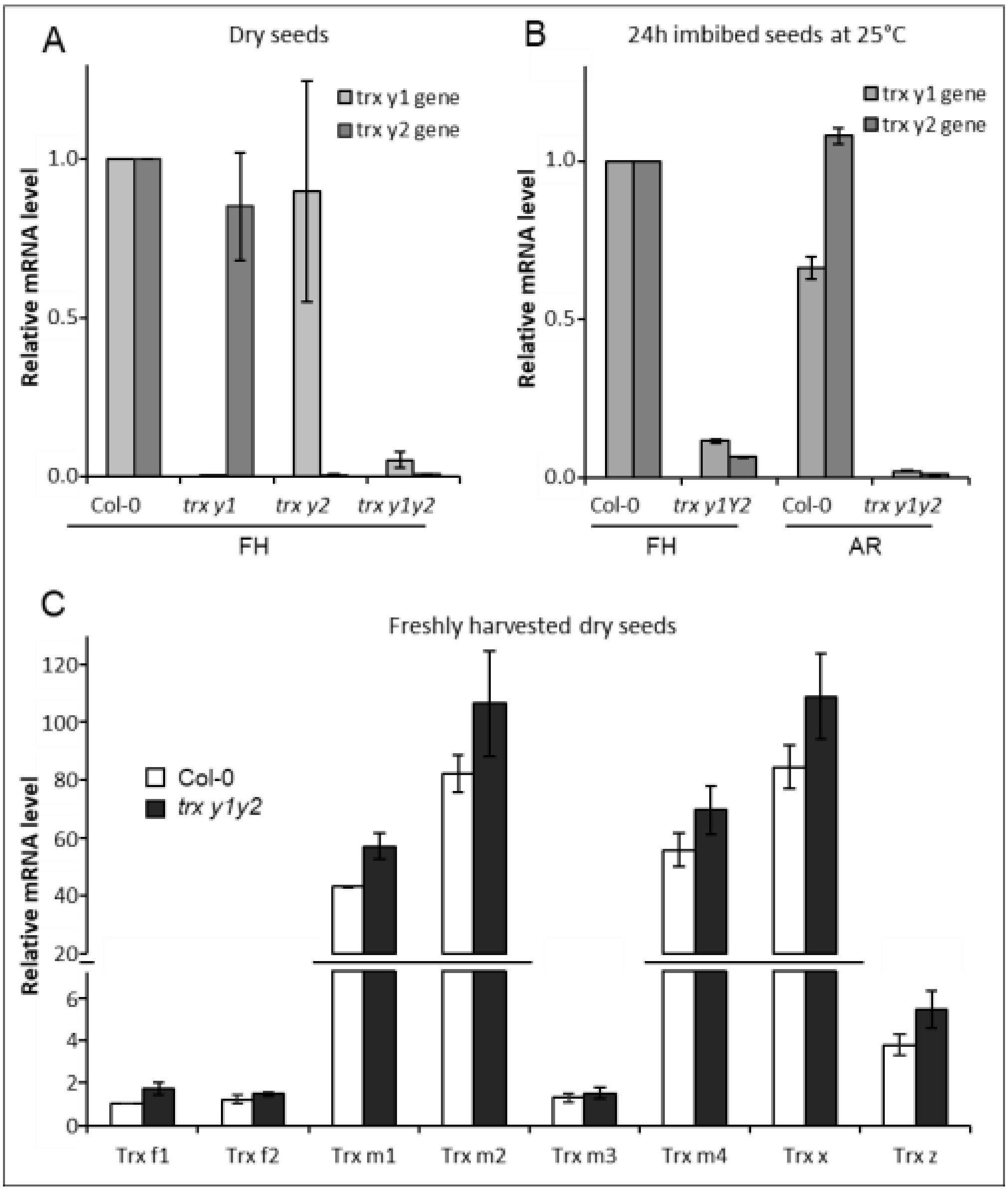

Figure S1. Transcript levels of Trxs genes in *trx y* mutant seeds. Expression levels of *TRXy1* and *TRXy2* genes were measured by RT-QPCR, (A) in freshly harvested dry seeds of Trxs y mutant lines (single and double mutants); (B) in imbibed seeds (with water for 24 h, in the dark, at 25 °C) of the double mutant *trx y1y2*, freshly harvested (FH) or 7 week after-ripened (AR). (C) mRNA levels of the other plastidial Trxs isoforms were also investigated by RT-QPCR, in freshly harvested dry seeds of the double mutant. Arbitrary units correspond to transcript levels normalized to *PP2A* constitutive gene in Col-0 (FH) in (A) and (C) and to At4g12590 seed constitutive gene in Col-0 (FH) sample in (B). Means ± SD of triplicate experiments are shown.

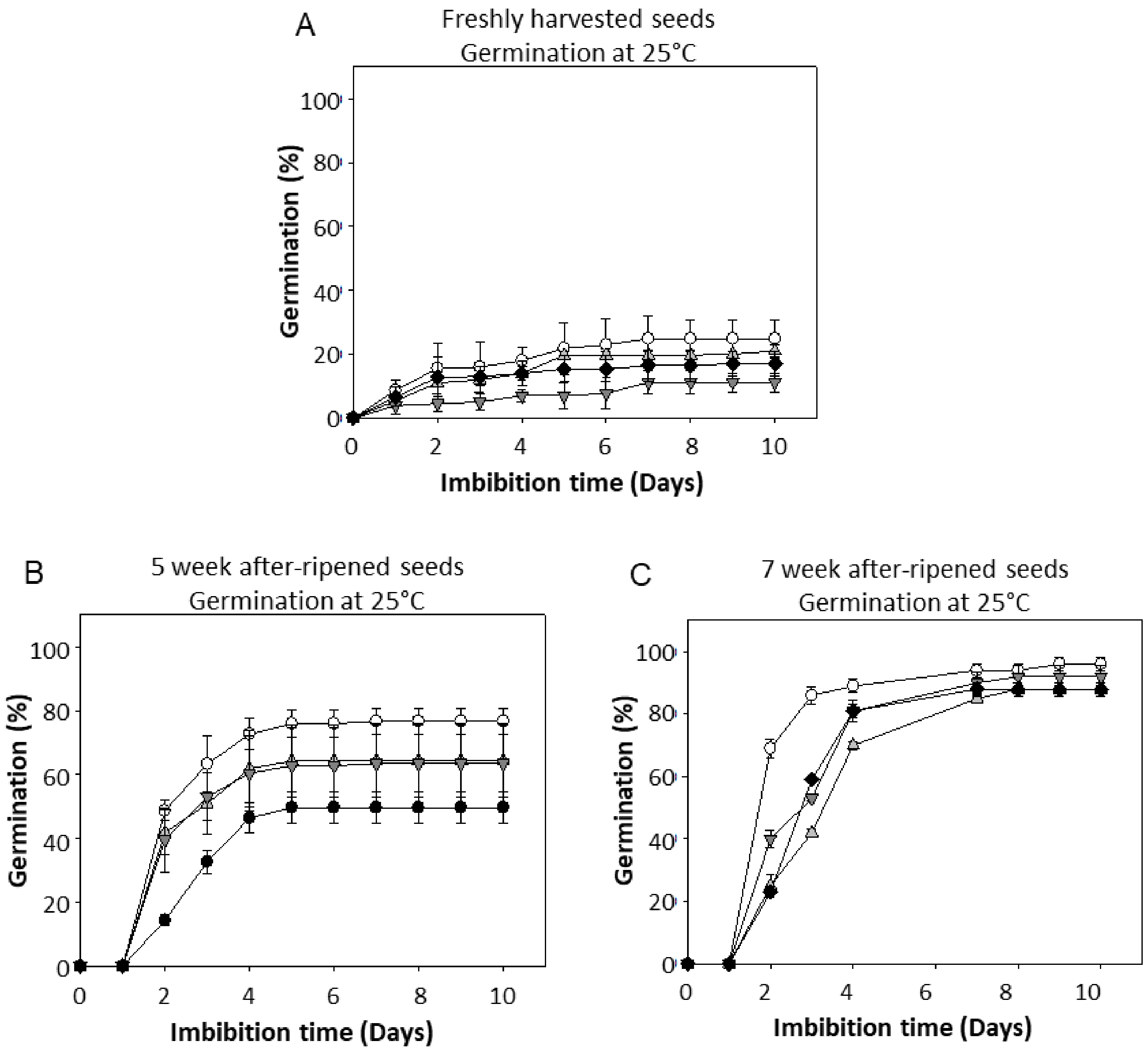

Figure S2. Germination of *trx y* mutant seeds along after-ripening. Germination of freshly harvested seeds (A), or 5 or 7 week after-ripened seeds (B and C) at 25°C. Means ± SD from triplicate experiments (3 independent seed batches).

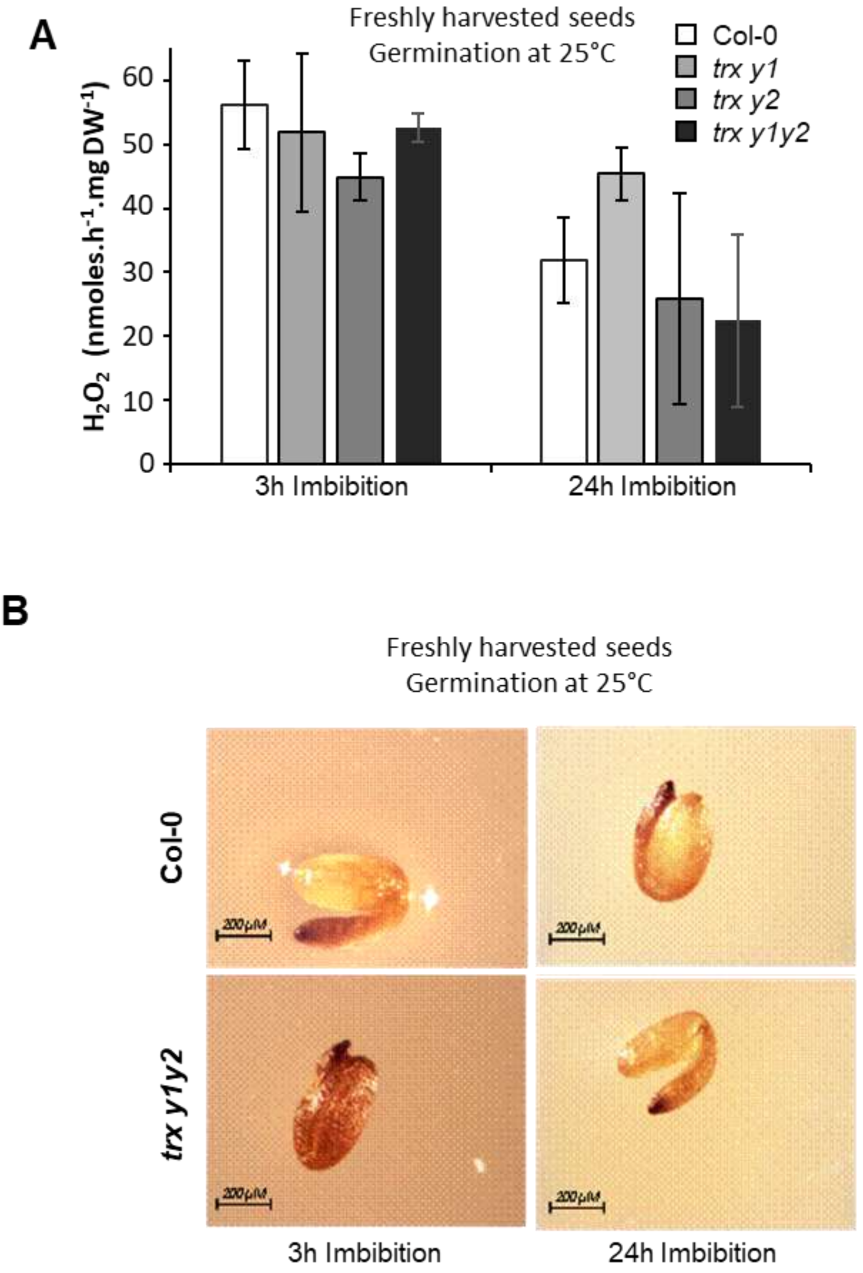

Figure S3. Hydrogen peroxide levels and superoxide anions accumulation patterns in *trx y* mutant seeds. (A) Production-diffusion of hydrogen peroxide by seeds. Freshly harvested seeds were imbibed for 3 or 24 h at 25°C in the dark, prior to incubation with scopoletin. Scopoletin oxidation was monitored using fluorimetry. Concentrations of H_2O_2 diffused from the seeds during an incubation time of 30 min were calculated from a reference curve. Data correspond to means ± SD of triplicate experiments. (B) *In situ* staining of superoxide anions. Freshly harvested seeds were imbibed (as mentioned above) prior dissection. Intact embryos were stained with 1 mM NBT (nitroblue tetrazolium) for 30 min at 25°C in 10 mM Tris-HCl pH 7.5 and extensively washed prior observation with a binocular.

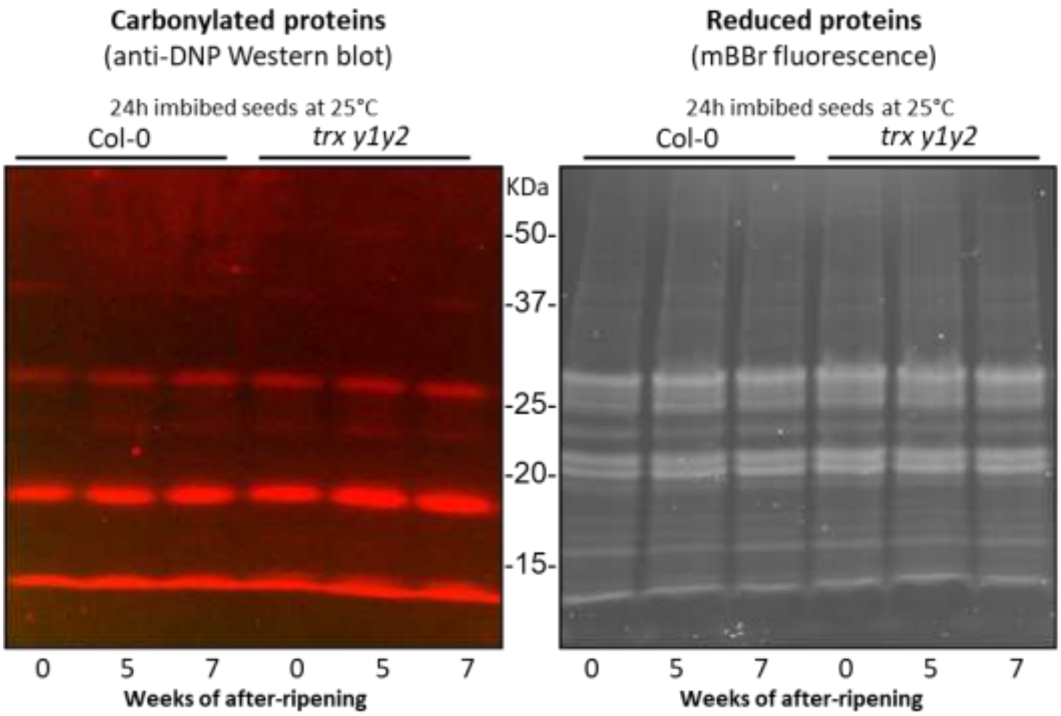

Figure S4. Redox status of seed proteins in Col-0 and the *trx y1y2* mutant. Proteins redox state was examined in freshly harvested seeds or in seeds after-ripened for 5 or 7 weeks after harvest. Protein redox state was examined using monobromobimane (mBBr) and dinitrophenyl hydrazine (DNPH). The mBBr probe fluoresces following covalent interaction with SH protein groups and allows the detection of reduced proteins. DNPH is a compound that reacts with carbonyl groups and allows the detection of proteins oxidized by carbonylation. Protein extractions were performed on seeds water-imbibed for 24 h, in the dark, at 25 °C. Left panel: Extent of protein oxidation as evidenced by carbonyl groups derivatization with DNPH. Right panel: Extent of protein reduction as evidenced by labelling of protein thiols with mBBr.

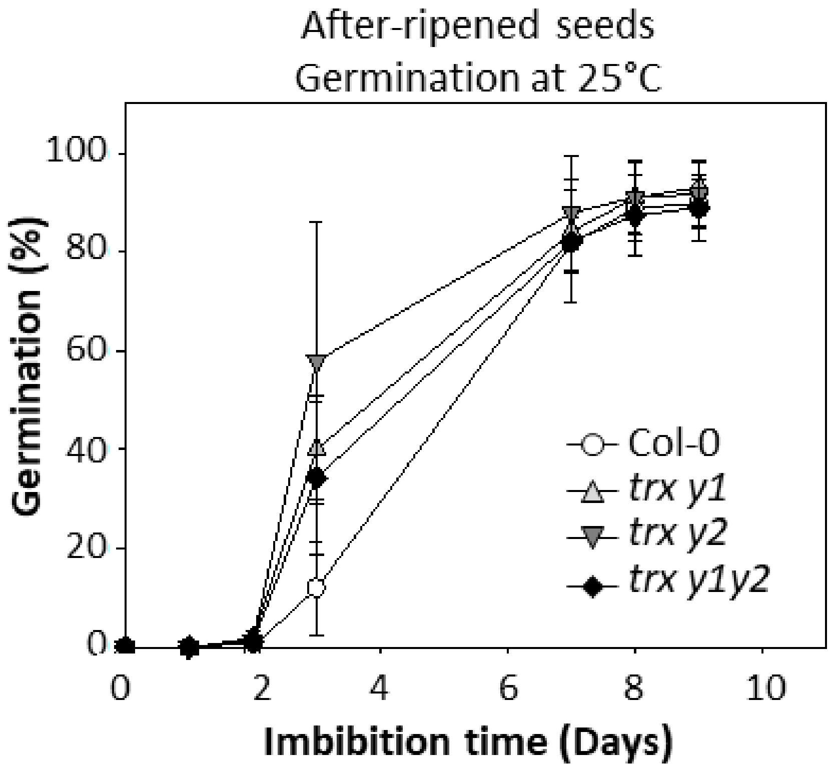

Figure S5. Germination of *trx y* mutant seeds under oxidative stress. Oxidative stress conditions for germination were imposed by exogenous application of methylviologen (10 µM). After-ripened seeds (8 weeks) were tested for germination at 25°C, in the dark. Technical triplicates were averaged. Bars indicate SD of the mean.

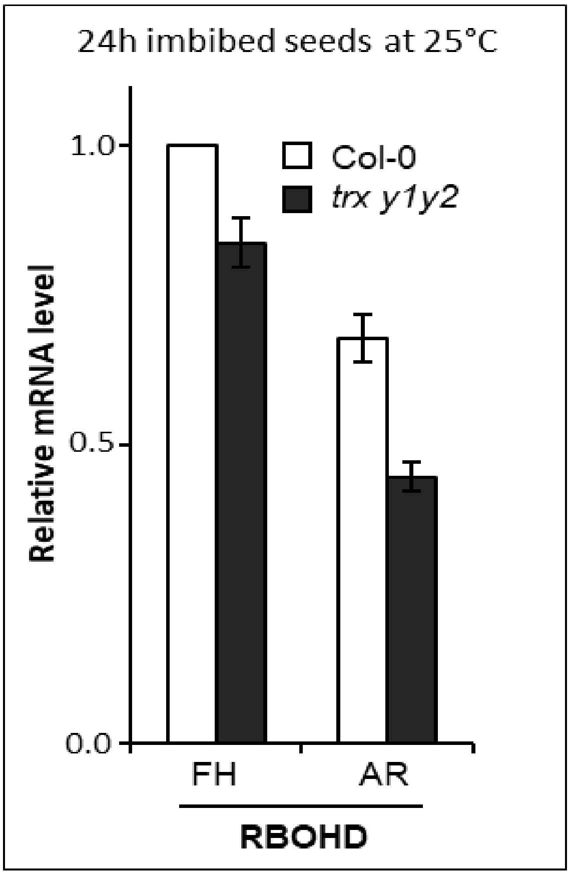

Figure S6. Expression level of *RBOHD* gene in the *trx y1y2* mutant seeds. Transcript abundance of RBOHD was examined using RT-QPCR in freshly harvested (FH) and 7 week after-ripened (AR) seeds, imbibed for 24 h, in the dark, at 25 °C. Transcript levels were normalized to the expression of At4g12590 seed constitutive gene and arbitrary unit was assigned to the expression level in Col-0 (FH) sample. Means ± SD from duplicate experiments.

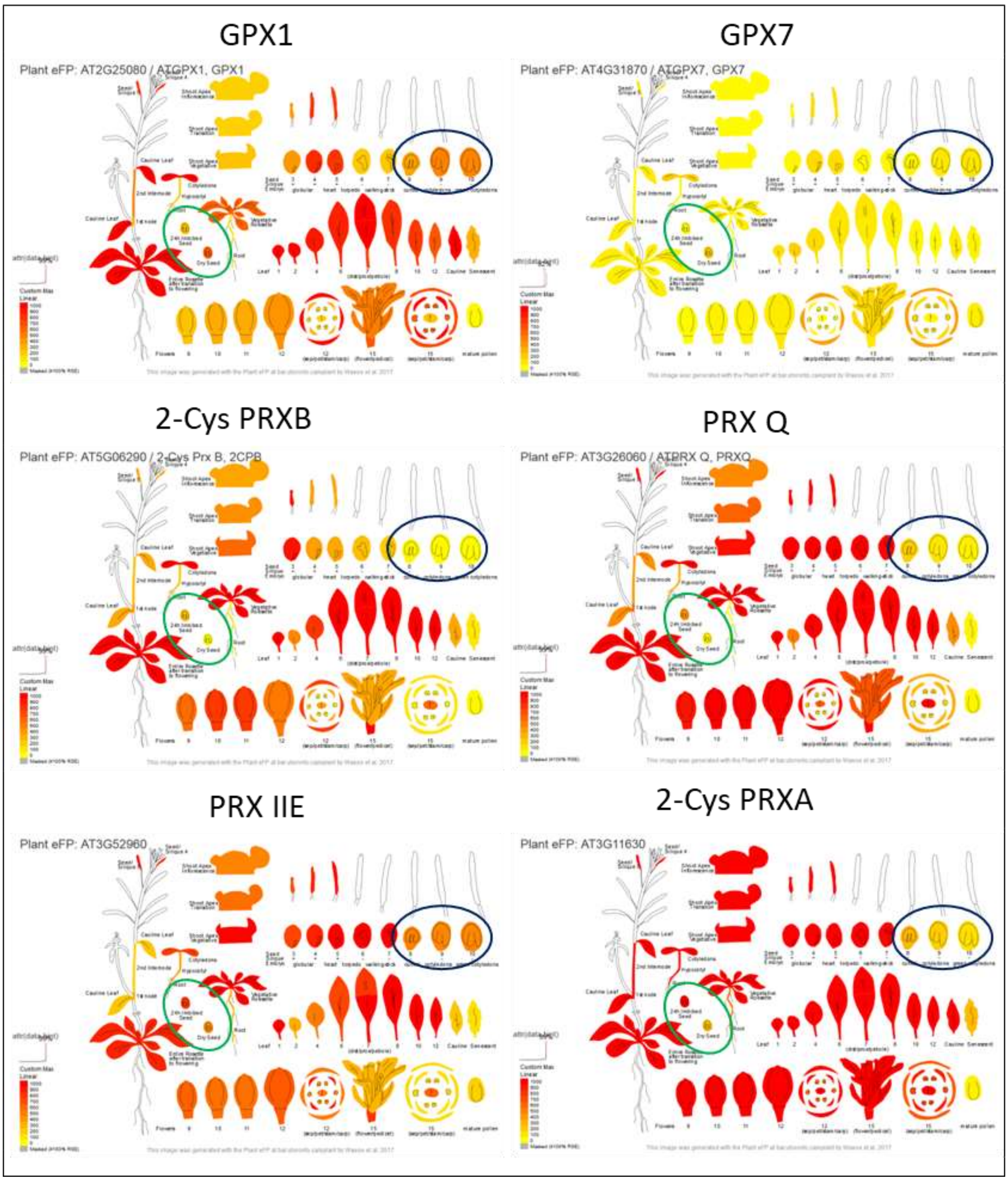

Figure S7. mRNA levels of peroxiredoxins (plastid isoforms) in Arabidopsis. Microarray data were obtained from the « Arabidopsis eFP browser » website (http://bar.utoronto.ca/efp2/Arabidopsis/Arabidopsis_eFPBrowser2.html). Late seed development stages and dry / imbibed seeds are highlighted by blue and green circles, respectively. Colour scale corresponds to the "absolute" expression level for a gene in each tissue, which is directly compared to the highest signal recorded for the given gene, with low levels of expression coloured in yellow and high levels coloured in red.
